# Increased PRR14 and VCAM-1 level in serum of patients with Parkinson's disease

**DOI:** 10.3389/fneur.2022.993940

**Published:** 2022-09-30

**Authors:** Huimin Zheng, Tai Wang, Changhe Shi, Liyuan Fan, Yun Su, Yu Fan, Xinwei Li, Jing Yang, Chengyuan Mao, Yuming Xu

**Affiliations:** ^1^Department of Neurology, The First Affiliated Hospital of Zhengzhou University, Zhengzhou University, Zhengzhou, China; ^2^The Academy of Medical Sciences of Zhengzhou University, Zhengzhou University, Zhengzhou, China; ^3^Henan Key Laboratory of Cerebrovascular Diseases, The First Affiliated Hospital of Zhengzhou University, Zhengzhou University, Zhengzhou, China; ^4^NHC Key Laboratory of Prevention and Treatment of Cerebrovascular Diseases, The First Affiliated Hospital of Zhengzhou University, Zhengzhou University, Zhengzhou, China; ^5^Department of Neurology, Nanyang Central Hospital, Nanyang, China; ^6^Institute of Neuroscience, Zhengzhou University, Zhengzhou, China

**Keywords:** Parkinson's disease, proline-rich protein 14, vascular cell adhesion molecule-1, soluble CD163, serum

## Abstract

**Background:**

Regarding the complexity of Parkinson's disease (PD), the identification of reliable biomarkers is of great significance for improving the accuracy of diagnosis and monitoring disease progression. Recently, some studies suggested that serum proline-rich protein 14 (PRR14), vascular cell adhesion molecule-1 (VCAM-1), and soluble CD163 (sCD163) factors may be associated with PD, even as potential biomarkers. However, the role of these serum factors is still unclear.

**Objectives:**

This study aimed to explore the alterations of serum PRR14, VCAM-1, and sCD163 levels during PD progression, and their association with disease-related variables of PD.

**Methods:**

We performed the assessment of scale tests and the detection of serum samples in patients with PD (*n* = 100) and healthy controls (HCs, *n* = 100). Furthermore, we investigated the association between serum factors and sex, cognitive impairments, H&Y (Hohn and Yahr), age at onset (AAO), and other variables in patients with PD.

**Results:**

Patients with PD exhibited increased PRR14 and VCAM-1 serum levels compared with HCs. No significant differences were found in serum levels of sCD163. Subgroup analysis uncovered increased VCAM-1 in the female and male subgroups (PD and HCs). Among patients with PD, decreased PRR14 and increased VCAM-1 were associated with severer cognitive impairments and severer PD (H&Y), respectively. Bivariate correlation analysis revealed that there was a positive correlation between VCAM-1 and AAO.

**Conclusions:**

Increased serum levels of PRR14 and VCAM-1 suggest that inflammation and defective autophagy may play vital roles in the pathogenesis of PD. However, the potential mechanisms remain to be elucidated.

## Introduction

Parkinson's disease (PD) is a common neurodegenerative disorder characterized by the degeneration of the nigral dopaminergic (DA) neurons and clumped Lewy bodies. Patients with PD pathologically manifest aggregated α-synuclein (α-syn) in the central nervous system (CNS) and peripheral autonomic nervous system (1, 2). It was difficult to precisely diagnose and discriminate against PD owing to its similar clinical performance to other common neurodegenerative diseases (3). Considerable efforts have been made to identify reliable biomarkers for serum samples. Notably, previous studies had found that alterations in serum proline-rich protein 14 (PRR14), vascular cell adhesion molecule-1(VCAM-1), and soluble CD163 (sCD163) levels, were probably linked with the constipation status (4), the severity of disease (5), the cognition levels (6), and so on in patients with PD. Moreover, PRR14 was linked with the protection of DA neurons and the clearance of aggregated α-syn (4, 7), and the ongoing neuroinflammatory processes were correlated with the VCAM-1 and sCD163 in PD (5, 6). However, there is a lack of reliable evidence regarding whether their serum levels can sensitively discriminate PD from healthy controls (HCs).

A compelling etiology of PD was that impaired autophagy could not dump the aggregated α-syn in the brain sites, accelerating the deterioration of PD (8). In PD, the mammalian target of rapamycin (mTOR) is a typical autophagy-related signal pathway. Furthermore, increased mTOR protein expression levels were associated with the aggregation of α-syn by inhibiting autophagy in patients with PD (9, 10). In previous studies, PRR14 was regarded as an activator of the mTOR signal and was also used as a potential biomarker for the diagnosis of PD (4, 11). PRR14 is increased in the cerebrospinal fluid (CSF), serum, and plasma samples of patients with PD and animal models of PD (4, 7, 11, 12). Elevated serum PRR14 levels can inhibit autophagy and the clearance of α-syn in PD (10). Conversely, high serum PRR14 levels can also protect against the loss of DA neurons in PD (4). Among patients with PD, high serum PRR14 levels increased the risk of constipation (4). However, the study for serum PRR14 levels was sparse, and there is no other research to provide a profound understanding of the mechanisms of PRR14.

Another etiology of PD is the activation of microglia-induced neuroinflammation, which brings out the aggregated α-syn and defective nigral DA neurons in the CNS (13, 14). VCAM-1, as a distinct hint of the monocyte activation signal, impaired the blood–brain barrier (BBB) function and promoted neuroinflammation and loss of DA neurons (7). The latest *in vitro* studies found that genetic ablation of VCAM-1 can ameliorate neuroinflammation (15). However, decreased VCAM-1 was found in the 6-hydroxydopamine (6-OHDA) animal models of PD (16). For patients with PD, higher serum VCAM-1 level was correlated with worsening motor symptoms and disease severity, which provides further evidence to monitor PD progression (5). In addition, as another indicator of monocyte activation, shedding of sCD163 enhances the α-syn uptake, and α-syn in turn induces sCD163 shedding by activating monocytes (6). Noteworthy, increased CD163 in the brain tissues was found in 1-methyl-4-phenyl-1,2,3,6-tetrahydropyridine-induced (MPTP) animal models of PD (17), while Calvello et al. found the converse results (18). For patients with PD, sCD163 in CSF and serum positively correlated with CSF levels of α-syn and cognitive deficits, which supported that sCD163 plays a vital role in PD (6). However, rare studies showed whether VCAM-1 and sCD163 were associated with phenotypic related variables in PD, and their alterations of them were still discrepant in clinical samples (6, 7).

In this study, we identified alterations in serum PRR14 and VCAM-1 levels of patients with PD. Furthermore, we investigated the associations between altered factors and age at onset (AAO), Hohn and Yahr (H&Y), sex, cognitive impairments, and other variables to provide further insights into the pathogenesis of PD.

## Patients and methods

### Patient recruitment

This study randomly enrolled 100 patients with sporadic PD and 100 age- and sex-matched healthy individuals. Patients with PD were clinically diagnosed according to the Movement Disorders Society Clinical Diagnostic Criteria for Parkinson's disease (19). Patients with PD were recruited from the outpatient clinic and ward in the Department of Neurology, The First Affiliated Hospital of Zhengzhou University from 2018 to 2021. Simultaneously, healthy control subjects were randomly recruited from community-dwelling individuals, who are without any evidence of neurodegenerative disease or parkinsonian symptoms. Specialized neurologists assessed all participants. For patients with PD, participants were excluded with the initial diagnosis of PD Dementia (PDD) and dementia with Lewy body (DLB) according to the recommended standards by the Movement Disorder Society (20, 21). Patients who were taking corticosteroids, non-steroidal anti-inflammatory drugs (NSAIDs), or had been diagnosed with other disorders, such as tumors, infectious diseases, cardiovascular disease, diabetes mellitus, or systemic autoimmune diseases, were excluded from this study. This study involving human participants was reviewed and approved by the Medical Research Ethics Committee of the First Affiliated Hospital of Zhengzhou University. Written informed consent was obtained from all participants before their inclusion in this study.

### Clinical assessments

We collected data on the demographic and clinical characteristics of patients with PD, including age, sex, age at PD onset, duration of PD, and level of education. The patients were evaluated to assess non-motor and motor symptoms during their “off state” using the Movement Disorder Society-Unified Parkinson's Disease Rating Scale (MDS-UPDRS). Simultaneously, the severity and stage of PD were obtained from the modified H&Y staging scale, and patients with PD were divided according to previous studies (22, 23). In detail, patients with PD with H&Y stage 1.0–2.0 were recognized as mild groups. H&Y stage 2.5–3.0 were divided into moderate groups, and H&Y stage 4.0–5.0 were severe groups. All participants underwent Mini-Mental State Examination (MMSE) assessment, accompanied by their level of education, to discern cognitive impairment (24). In detail, the established MMSE cutoff scores for the cognitive impairment of the participants are <17 for illiterate, <20 for individuals with 1–6 years of education, and <24 for individuals with 7 or more years of education (24). The cut-off values of disease duration and AAO were set according to the mean to distinguish discrete subgroups. The treatment of antiparkinsonian medication was also recorded. Simultaneously, levodopa-equivalent daily doses (LEDDs) were calculated as previously described (25). In particular, owing to the bad prognosis of the postural instability/gait difficulty (PIGD) group (26), we distinguished patients with PD with distinct clinical phenotypes, such as tremor dominant (TD), PIGD, and indeterminate (ID) phenotypes.

### Measurement of factors in the serum

In the morning, venous blood samples were collected after overnight fasting using vacuum tubes, which minimizes confounding factors, such as diet and circadian rhythm. The samples were centrifuged at 1,000 *g* for 10 min at 4°C, and serum was collected and stored at –80°C for subsequent analysis. Specifically, serum levels of PRR14 (Jianglaibio, China) were detected using an Enzyme-linked Immunosorbent Assay (ELISA) kit, in accordance with the manufacturer's instructions. In addition, we used the Magnetic Luminex Assay-Human Premixed Multi-Analyte kit to determine the circulating levels of VCAM-1 and sCD163 according to the manufacturer's instructions (R&D Systems, Minneapolis, MN, USA). The minimum detectable dose of PRR14 was 2.5 ng/ml, VCAM-1 was 2,819.6 pg/ml, and sCD163 was 1,874.8 pg/ml.

### Statistical analysis

All data were analyzed using the SPSS software (version 21.0; Armonk, NY, USA). Individual characteristics are displayed as the mean ± standard deviation (SD) for normally distributed variables and as the median along with the interquartile range for variables with skewed distributions. Fisher's *t*-test was used to compare binary variables. For continuous variables, the Shapiro–Wilk test was used to test the normality of the sample distribution. The two groups (patients with PD vs. HCs) were compared using the Mann–Whitney *U*-test or Student's *t*-test when the statistics were non-normally and normally distributed, respectively. Multiple group comparisons were used the Kruskal–Wallis non-parametric test and one-way ANOVA for non-parametric and parametric distributions, respectively. Of note, the *post-hoc* test (LSD) test or Kruskal–Wallis test was used for pairwise comparisons after comparisons of multiple groups. Bivariate correlations using Spearman's correlation analysis were performed to examine the relationship between serum factors and disease-related variables. All statistical tests were two-tailed, and the threshold for statistical significance was set at *P* < 0.05. Analyses were performed using the GraphPad Prism software (version 8.0.2; San Diego, CA, USA).

## Results

### Demographic and clinical characteristics

A total of 200 participants were enrolled in the study, including patients with PD (*n* = 100) and HCs (*n* = 100). Basic information about the participants in both groups is summarized in Table 1. No significant differences in age or sex were observed between the patient and the control groups. Among the 100 patients with PD, men occupied the major position (female/male =37/63), along with a mean age of 59.45 ± 8.58 (mean ± SD) years. The mean duration of illness was 4.44 ± 3.17 years, and the mean AAO was 54.76 ± 9.03 years. For PD participants, the UPDRS III and MMSE scores were 39.31 ± 19.16 (7–116) (mean ± SD (range) and 24.30 ± 5.50 (6–30), respectively. The years of education in patients with PD were 9.46 ± 4.44 (0–16). The percentage of individuals taking antiparkinsonian medication was 80% in patients with PD. Among the medicated patients, the percentage of using L-dopamine and dopamine agonists was 86.25 and 48.75%, respectively. Especially, the LEDDs were 381.70 ± 339.99 (0–2,060), along with the denoted H&Y stage was 2.10 ± 0.85 (1–5).

**Table 1:** Demographic and clinical characteristics of patients with Parkinson's disease and HCs.

**Variable**	**PD patients (*n* = 100)**	**HCs (*n* = 100)**	***p***
Gender, *n* (female/male)	37/63	50/50	0.064
Age, y, mean ± SD	59.45 ± 8.58 8.58246 ± 9.75	57.44 ± 7.36	0.079
AAO, y, mean ± SD	54.76 ± 9.03	NA	NA
Duration of PD, y, mean ± SD (range)	4.44 ± 3.17 (1–17)	NA	NA
UPDRS I, mean ± SD (range)	8.18 ± 5.73 (0–27)	NA	NA
UPDRS II, mean ± SD (range)	13.80 ± 7.77 (1–42)	NA	NA
UPDRS III, mean ± SD (range)	39.31 ± 19.16 (7–116)	NA	NA
H&Y, mean ± SD (range)	2.10 ± 0.85 (1–5)	NA	NA
MMSE, mean ± SD (range)	24.30 ± 5.50 (6–30)	NA	NA
Years of education, mean ± SD (range) education	9.46 ± 4.44 (0–16)	NA	NA
Use of antiparkinsonian medication, %	80%	NA	NA
Use of L-dopamine, %	86.25%	NA	NA
Use of dopamine agonist, %	48.75%	NA	NA
LEDDs (mg/d), mean ± SD (range)	381.70 ± 339.99 (0–2,060)	NA	NA

Categorical variables were compared by using Pearson's Chi-square test. Continuous variables in normal distribution and homogeneity of variance were compared by using the two-sample t-test, otherwise, the Mann–Whitney test was performed. PD, Parkinson's Disease; HCs, healthy controls; AAO, age at PD onset; UPDRS, Unified Parkinson's Disease Rating Scale; H&Y, Hoehn and Yahr scale; LEDDs, levodopa-equivalent daily doses; MMSE, Mini-Mental State Examination; NA, not available; n, number.

### Altered factors in the serum

**Figure 1:**
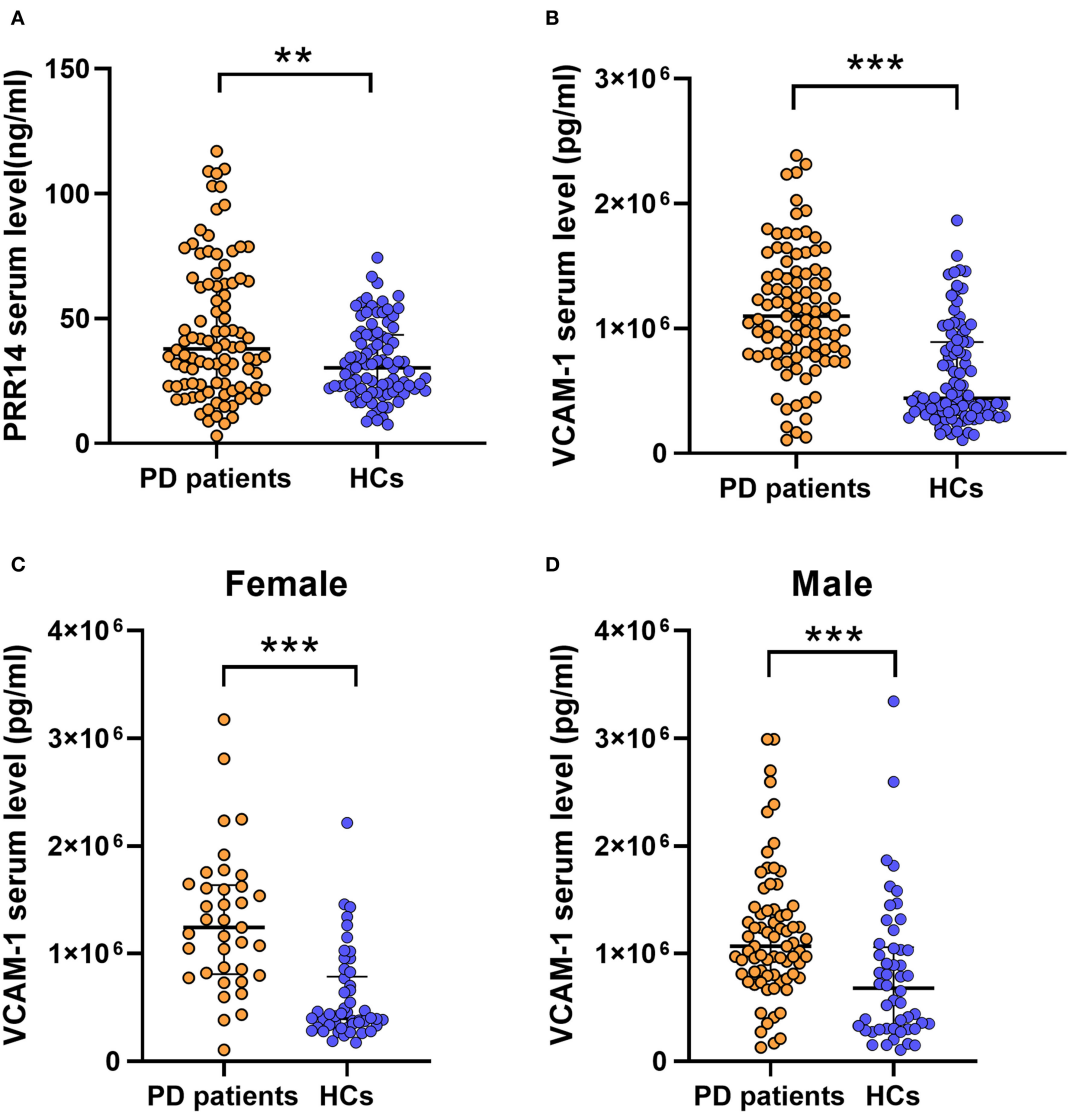
Significantly serum factors implies that inflammation and defective autophagy might play a vital role in the pathophysiology of PD. Higher serum PRR14 **(A)** and VCAM-1 **(B)** levels were observed in PD patients compared with HCs. In sex subgroup analysis, PD patients exhibited increased VCAM-1 serum levels compared with HCs both in female subgroups **(C)** and male subgroups **(D)**. **P < 0.01; ***P < 0.001, Mann–Whitney *U* test. PD, Parkinson's disease; HCs, healthy controls; PRR14, proline-rich protein 14; VCAM-1, Vascular cell adhesion molecule-1.

For 200 participants, significant results showed that higher serum PRR14 levels (37.82 vs. 30.26 ng/ml, *P* = 0.009; Figure 1A; Table 2) and VCAM-1 levels (1,098,200.00 vs. 440,188.30 pg/ml, *P* = 0.001; Figure 1B; Table 2) were observed in patients with PD than in HCs. However, serum levels of sCD163 showed no significant difference between patients with PD and HCs (269,202.18 vs. 191,443.60 pg/ml, *P* = 0.114; Supplementary Figure S1A; Table 2).

**Table 2:** Serum PRR14 and VCAM-1 levels altered in patients with PD vs. HCs.

**Variable**	**PD patients (*n* = 100)**	**HCs (*n* = 100)**	***p***
PRR14, ng/ml, median (QL–QU)	37.82(22.78–64.16)	30.26(21.22–43.45)	0.009**
VCAM-1, pg/mL, median (QL–QU)	1,098,200.00 (797,190.05–1,443,700.00)	440,188.30 (305,452.80–890,782.00)	<0.001***
sCD163, pg/mL, median (QL–QU)	269,202.18 (135,233.85–339,920.24)	191,443.60 (97,426.43–335,048.73)	0.114

Elevated PRR14 and VCAM-1 serum levels among patients with PD reached statistically significant compared with HCs. However, sCD163 was unremarkable in our study. Continuous variables in normal distribution and homogeneity of variance were compared by using the two-sample t-test, otherwise, the Mann–Whitney test was performed. **P < 0.01; ***P < 0.001. PD, Parkinson's disease; HCs, healthy controls; PRR14, proline-rich protein 14; VCAM-1, vascular cell adhesion molecule-1; sCD163, soluble CD163; NA, not available; n, number.

### Subgroup analyses and correlation analyses among patients with PD

**Figure 2:**
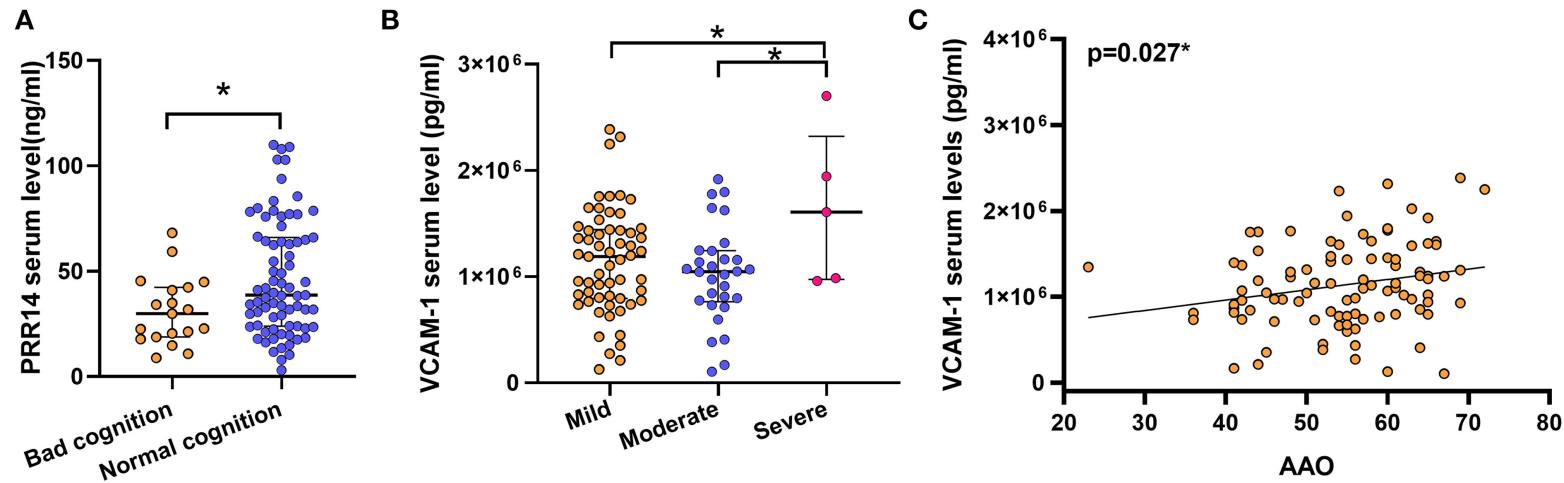
Probable prognostic variables affecting altered factors were uncovered in the subgroup analyses. Lower serum PRR14 levels were linked with severer cognitive impairments **(A)**. *P < 0.05, Mann–Whitney *U* test. Increased VCAM-1 was associated with severer PD **(B)**. *P < 0.05, Kruskal–Wallis test. Possible relationship between the altered serum factors and clinical related variables were examined by Bivariate association analysis. The results revealed that a later AAO was associated with higher serum VCAM-1 levels **(C)**. *P < 0.05, Spearman correlation test. PD, Parkinson's disease; PRR14, proline-rich protein 14; VCAM-1, Vascular cell adhesion molecule-1; AAO, Age at onset.

For further analysis, we divided the patients with PD into discrete subgroups according to sex, cognitive impairment status, H&Y stage, and other variables. In sex subgroup analysis, patients with PD exhibited increased VCAM-1 serum levels compared with HCs in both female subgroups (*P* < 0.001; Figure 1C; Supplementary Table 1) and male subgroups (*P* = 0.001; Figure 1D; Supplementary Table 1). And the PPR14 and sCD163 serum levels (Supplementary Figures S1B–E; Supplementary Table 1) were observed without some sex differences throughout the sex subgroup analysis. For two groups (with or without cognitive impairment) based on the MMSE test score and the level of education, it proved that lower serum PRR14 levels were linked with severer cognitive impairments (*P* = 0.048; Figure 2A; Supplementary Table 2). As for three distinctive groups (Mild, Moderate, and Severe groups) defined from the H&Y stage, increased VCAM-1 was associated with severer PD (H&Y) (*P* =0.036; Figure 2B; Supplementary Tables 3, 4). Further pairwise comparisons implied that there was a significance in serum VCAM-1 level between the Mild groups vs. Severe groups or Moderate groups vs. Severe groups (*P*=0.034; Figure 2B; Supplementary Table 4). However, other residual subgroup analyses did not reveal a significant difference (Supplementary Tables 5–7), regardless of the potential tendency.

Apart from subgroups analysis, bivariate correlation analysis only revealed that there was a positive correlation between VCAM-1 and AAO, but not with other disease-related variables (*r* = 0.252, *P* = 0.008; Figure 2C; Table 3). Nevertheless, serum levels of PRR14 were irrelevant to other clinical variables (Table 3). These novel findings need to be confirmed by further experiments.

**Table 3:** Correlation between serum PRR14 or VCAM-1 and some clinical variables in patients with PD.

**Variable**	**PRR14 (ng/ml)**		**VCAM-1 (pg/mL)**	
	**Rs**	***P***	**Rs**	***P***
AAO	0.063	0.540	0.227	0.027*
Duration of PD	0.047	0.648	0.128	0.216
UPDRS I	−0.087	0.402	0.049	0.640
UPDRS II	−0.094	0.362	0.173	0.094
UPDRS III	0.034	0.743	0.165	0.110
LEDDs	0.119	0.246	0.006	0.957
H&Y	0.112	0.278	0.074	0.482
MMSE	0.117	0.256	−0.071	0.497

Binary variables correlation analysis detected the association between serum PRR14 or VCAM-1 and other variables in patients with PD. It revealed that there is a significant positive correlation between VCAM-1 and AAO. *P < 0.05, Spearman correlation test. PD, Parkinson's disease; PRR14, proline-rich protein 14; VCAM-1, vascular cell adhesion molecule-1; Rs, correlation coefficient; AAO, age at onset; LEDDs, Levodopa-equivalent daily doses; H&Y, Hohn and Yahr; MMSE, Mini-Mental State Examination.

## Discussion

Based on the similar clinical properties of PD compared with other neurodegenerative diseases, it is significant to find an accessible biomarker to effectively diagnose PD with high sensitivity and specificity. An accumulating body of research has focused on alterations in serum factors which plays a crucial role in the pathophysiology of PD (10, 14, 27, 28). Previous studies have shown inconsistent results for factors (PRR14, VCAM-1, and sCD163) that are linked with PD (4, 6, 29). Generally, PRR14 can slow down the clearance of aggregated α-syn or protect the DA neurons (4, 7), while VCAM-1 and sCD163 were involved with the ongoing neuroinflammatory processes in PD (5, 6). In this study, we found that serum levels of PRR14 and VCAM-1 increased in patients with PD. However, no significant difference was observed in the serum sCD163 levels. Furthermore, we found that decreased PRR14 and increased VCAM-1 were linked with severer cognitive impairments and severer PD (H&Y), respectively. As for the bivariate correlation analysis, we uncovered a positive correlation between VCAM-1 and AAO.

Depending on two separable modular domains, PRR14 bridged the nuclear lamina or nuclear membrane and heterochromatin, which plays a role in the potentiation of mTOR signal and inhibition of autophagy (30, 31). As a well-known oncogene, many studies have demonstrated the upregulation of PRR14 in breast cancer (32), lung cancer (33), colon cancer (31), and so on. This sign was regarded as carcinogenesis and progression, which relies on the activation of the PI3-kinase/Akt/mTOR pathway (4). This common pathway also participates in PD and other degenerative diseases *via* autophagy (10, 34). Of note, as an activator for the mTOR pathway, increased PRR14 is detected in the CSF, serum, and plasma samples of patients with PD and relevant animal models (4, 7, 11, 12), which may result in the suppression of autophagy, accumulation of α-syn, and neuronal death (4). Conversely, some studies suggested that increased serum PRR14 levels were linked with the protection of DA neurons (4). Interestingly, among patients with PD, high serum PRR14 levels increased the risk of constipation (4). In our study, increased PRR14 was similar to other studies in patients with PD (4). Noteworthy, our serum PRR14 levels for HCs were different compared with previous studies, which could attribute to the regional distinction and difference in the size of samples (4). As for the subgroup analysis, we first found that lower PRR14 levels were associated with severer cognitive impairments (*P* = 0.048; Figure 2A, Supplementary Table 2). Further explorations are required to fully understand the increased PRR14 effect on cognitive impairments during PD.

Vascular cell adhesion molecule-1is a vascular inflammation factor that plays an important role in BBB dysfunction and neoangiogenesis (5, 35). With the initiation of inflammation, activated microglia and astrocytes release typical pro-inflammatory cytokines to disrupt the BBB. Hence, VCAM-1 was released from brain endothelial cells (BECs), resulting in the migration of peripheral immune cells from the peripheral blood into the CNS (5, 36–38). Thus, neuroinflammation was aggravated in patients with PD (36). Increased VCAM-1 was demonstrated in the serum and plasma of patients with PD (7, 29), and in the CSF of diabetes patients (39). Interestingly, CSF levels of VCAM-1 positively correlated with BBB permeability (39), and VCAM-1 can directly increase BBB permeability (35). Genetic ablation of VCAM-1 in BECs can ameliorate neuroinflammation *via* the VCAM-1-very late antigen 4 (VLA4)-axis (15). In a 6-OHDA animal model of PD, decreased VCAM-1 was found in the midbrain, instead of the striatum (16). Of note, higher VCAM-1 levels were positively linked with cognitive impairment in a large cohort of elderly participants (40). For patients with PD, higher serum VCAM-1 level was positively correlated with worsening motor symptoms (MDS-UPDRS score) and disease severity (H&Y stage), which supported that VCAM-1 is a potential biomarker (5, 7). In addition, higher VCAM-1 levels were found in fatigued patients with PD compared with non-fatigued patients with PD (29), and the factors were negatively correlated with the gray matter volume of some brain regions (left parahippocampus, cerebellum, etc.) in patients with PD, which uncovered the relationship between vascular inflammatory and brain atrophy during the neurodegenerative process in PD (5). In our study, serum VCAM-1 levels were increased in patients with PD. Subgroup analyses indicated that higher VCAM-1 was associated with severer PD (Mild groups vs. Severe groups; Figure 2B; Supplementary Tables 1–7) and later AAO (Figure 2C; Table 3). We speculated that later AAO of patients with PD had defective adaptive immune systems and were sensitive to stressors, which increased the risk of developing PD (5, 41). The mechanisms underlying our results remain to be explored in the future.

It is well-known that increased sCD163 is a distinct sign of monocyte activation, which is exclusively shed from the surface of monocytes and macrophages to produce soluble products (6, 42, 43). Notably, sCD163 shedding enhances α-syn uptake, and α-syn in turn induces sCD163 shedding by activating monocytes in myeloid cell lines and monocyte-derived macrophages, respectively (6). In typical MPTP PD animal models, CD163 was increased in the striatum and substantia nigra, while other studies displayed converse results (17). Corresponding studies suggested that sCD163 was a novel biomarker in PD because elevated CSF and serum of sCD163 were positively correlated with α-syn levels and cognitive impairments in patients with PD (6, 43). In addition, previous studies found that serum sCD163 levels were elevated in late PD women, but not in men, which implied the sex differences (6). However, our study showed no significant difference in serum sCD163 levels of patients with PD and HCs (Supplementary Figure S1A; Table 2). These discordant results could be attributed to regional differences or differences in sample sizes among the participants, which requires more evidence.

In conclusion, we found that serum levels of PRR14 and VCAM-1 were increased in patients with PD compared to HCs. Furthermore, decreased PRR14 was associated with severer cognitive impairments, and increased VCAM-1 was linked with severer PD and later AAO. However, our studies had some limitations, such as the small sample size (patients with PD and HCs), higher percentages of using antiparkinsonian medication (80%; Table 1), and lower education level, which may slightly influence the values of results. Therefore, future studies will increase the sample size and recruit more matched and general patients (the whole medication-naïve patients) and control subjects to gain a comprehensive understanding of the mechanisms of PD. Overall, these results suggest that the initiation of the inflammation cascade and defective autophagy might play a vital role in the pathophysiology of PD.

## Data availability statement

The datasets to this article are available from the corresponding author upon reasonable request.

## Ethics statement

The studies involving human participants were reviewed and approved by Medical Research Ethics Committee of the First Affiliated Hospital of Zhengzhou University. The patients/participants provided their written informed consent to participate in this study.

## Author contributions

HZ: designed the research project, organized the study, executed the research, designed the statistical analysis, and wrote the first draft. TW: executed the research and designed the statistical analysis. CS: executed the research, reviewed the manuscript, and provided funding. LF: organized the study and executed the statistical analysis. YS and XL: organized the study. YF: executed the statistical analysis and reviewed the statistics. JY: reviewed the manuscript. CM: designed the research project, reviewed the manuscript, and provided funding. YX: provided funding. All authors contributed to the article and approved the submitted version.

## Funding

This work was supported by the National Natural Science Foundation of China (Grant U1904207 and 91849115 to YX, Grant 81771290 and 81974211 to CS, Grant 82271277 and 81901300 to CM), Provincial Key Scientific and Technological Projects (Grant SBGJ202003020 to CM), the Innovative and Scientific and Technological Talent Training Project of Henan Province (Grant YXKC2021062 to CM), and the Non-Profit Central Research Institute Fund of Chinese Academy of Medical Sciences.

## Conflict of interest

The authors declare that the research was conducted in the absence of any commercial or financial relationships that could be construed as a potential conflict of interest.

## Publisher's note

All claims expressed in this article are solely those of the authors and do not necessarily represent those of their affiliated organizations, or those of the publisher, the editors and the reviewers. Any product that may be evaluated in this article, or claim that may be made by its manufacturer, is not guaranteed or endorsed by the publisher.

## Supplementary material

Supplementary Figure 1 For the alteration of PD patients and HCs groups, no significance was found in the serum sCD163 levels between two groups **(A)**. For sex subgroup analysis, PRR14 and sCD163 serum levels were observed without the sex differences among the female **(B,D)** and male subgroups **(C,E)**. Mann–Whitney *U* test. PD, Parkinson's disease; PRR14, proline-rich protein 14; sCD163, soluble CD163.

## Abbreviations

PD: Parkinson's disease
DA neurons: dopaminergic neurons
α-syn: α-synuclein
CNS: central nervous system
PRR14: proline-rich protein 14
VCAM-1: vascular cell adhesion molecule-1
sCD163: soluble CD163
HCs: healthy controls
mTOR: the mammalian target of rapamycin
CSF: cerebrospinal fluid
BBB: blood–brain barrier
6-OHDA: 6-hydroxydopamine
MPTP, 1-methyl-4-phenyl-1,2,3: 6-tetrahydropyridine
AAO: age at onset
H&Y: Hohn and Yahr
PDD: PD Dementia
DLB: dementia with Lewy body
NSAIDs: non-steroidal anti-inflammatory drugs
MDS-UPDRS: Movement Disorder Society-Unified Parkinson's Disease Rating Scale
MMSE: Mini-Mental State Examination
LEDDs: levodopa-equivalent daily doses
PIGD: postural instability/gait difficulty
ID: Indeterminate
TD: tremor dominant
ELISA: Enzyme-linked Immunosorbent Assay
LSD test: *post-hoc* test (LSD) test
BECs: brain endothelial cells
VLA4: very late antigen 4.
