# Supplementary material for: Increased PRR14 and VCAM-1 level in serum of patients with Parkinson's disease
Source: Front Neurol. 2022 Sep 30;13:993940. doi: 10.3389/fneur.2022.993940 (PMC9561935; doi:10.3389/fneur.2022.993940)
Supplement: Supplementary Figure 1 — For the alteration of PD patients and HCs groups, no significance was found in the serum sCD163 levels between two groups (A). For sex subgroup analysis, PRR14 and sCD163 serum levels were observed without the sex differences among the female (B,D) and male subgroups (C,E). Mann–Whitney U test. PD, Parkinson's disease; PRR14, proline-rich protein 14; sCD163, soluble CD163. [file Data_Sheet_1.docx]

Supplementary Material

# Supplementary Tables

**Supplementary Table 1.** Characteristics of female and male subgroups of PD patients

| **Variable** | **Sex** | **PD patients (n = 100)** | **HCs (n = 100)** | **p** |
| --- | --- | --- | --- | --- |
| PRR14, ng/ml, median (QL–QU) | Male | 43.50(23.64–75.96) | 34.64 (23.17–51.44) | 0.088 |
|  | Female | 31.76(19.75–41.34) | 25.75(20.38–39.34) | 0.492 |
| VCAM-1, pg/mL, median (QL–QU) | Male | 1002719.82  (790516.44–1350400.00) | 609306.05  (300569.80–1034250.00) | < 0.001*** |
|  | Female | 1215550.00  (802560.78–1619950.00) | 369458.35  (282461.78–405781.80) | < 0.001*** |
| sCD163, pg/mL, median (QL–QU) | Male | 276942.69  (153335.07–332545.49) | 191443.60  (118362.10–373023.80) | 0.283 |
|  | Female | 220030.97  (100177.04–395427.90) | 196172.30  (87593.42–331481.00) | 0.424 |

Altered PRR14, VCAM-1, and sCD163 was postulated as the sex-associated. Hence, we divided PD patients and HC subjects into distinct groups base on the sex. Male PD patients = 63, Female PD patients = 37. Male control patients = 50, Female control subjects = 50. We founded that PD patients exhibited increased VCAM-1 serum levels compared with HCs both in female subgroups and male subgroups. Continuous variables in normal distribution and homogeneity of variance were compared by using the two-sample t test, otherwise, the Mann–Whitney test were performed. ** P < 0.05; **P < 0.01; ***P < 0.001.*

**Abbreviation:** PD, Parkinson's disease; HCs, healthy controls; PRR14, proline-rich protein 14; VCAM-1, vascular cell adhesion molecule-1; sCD163, soluble CD163; NA, not available; n, number.

**Supplementary Table 2.** Serum factors and clinical features according to with or without cognitive impairment in patients with PD

| **Variable** | **PD patients with**  **cognitive impairment (n=23)** | **PD patients without**  **cognitive impairment (n=77)** | **p** |
| --- | --- | --- | --- |
| Gender, n (female/male) | 11/12 | 26/51 | 0.220 |
| Age y, mean ± SD | 61.22 ± 8.31 | 58.57 ± 9.12 | 0.358 |
| AAO, y, mean ± SD | 56.04 ± 7.64 | 54.38 ± 9.42 | 0.517 |
| Duration of PD, y, mean ± SD (range) | 5.22 ± 4.09 (1–15) | 4.21 ± 2.83 (1–17) | 0.533 |
| LEDDs (mg/d), mean ± SD (range) | 394.13 ± 310.52 (0–950) | 377.99 ± 350.15 (0–2060) | 0.705 |
| H&Y, mean ± SD (range) | 2.33 ± 1.02 (1–5) | 2.03 ± 0.79 (1–5) | 0.261 |
| PRR14, ng/ml, median (QL–QU) | 29.80(18.70–42.28) | 38.62(23.90–65.99) | 0.028* |
| VCAM-1, pg/mL, median (QL–QU) | 1172800.00  (796689.15–1485000.00) | 1101550.00  (796792.36–1446325.00) | 0.851 |

In subgroup analyses for cognitive condition, we divided PD patients into two groups basing on the MMSE test score and the level of education. We found that serum VCAM-1 levels were irrelevant to the impaired cognition, but higher serum PRR14 levels were linked with severer cognitive impairment. Categorical variables were compared by using the Pearson Chi–square test. Continuous variables in normal distribution and homogeneity of variance were compared by using the two-sample t test, otherwise, the Mann–Whitney test were performed. ** P < 0.05; **P < 0.01; ***P < 0.001.*

**Abbreviation:** PD, Parkinson's disease; AAO, age at onset; H&Y, Hoehn and Yahr scale; LEDDs, levodopa-equivalent daily doses; MMSE, Mini-Mental State Examination; VCAM-1, vascular cell adhesion molecule-1; PRR14, proline-rich protein 14; n, number.

**Supplementary Table 3.** Serum factors and clinical features according to H&Y stage in patients with PD

| **Variable** | **PD patients (n = 100)** | | |  |  |
| --- | --- | --- | --- | --- | --- |
|  | **Mild (n = 62)** | **Moderate (n = 33)** | **Advanced (n = 5)** | **HCs**  **(n = 100)** | **p** |
| Age y, mean ± SD | 58.23 ± 9.08 | 60.06 ± 9.04 | 65.20 ± 3.70 | 57.44 ± 7.36 | 0.120 |
| AAO, y, mean ± SD | 54.61 ± 9.27 | 54.64 ± 9.20 | 57.40 ± 4.83 | NA | 0.886 |
| Duration of PD, y, mean ± SD (range) | 3.66 ± 2.56 (1–13) | 5.39 ± 3.45 (1–17) | 7.80 ± 4.71 (2–15) | NA | 0.004** |
| UPDRS I, mean ± SD (range) | 7.32 ± 6.10 (0–26) | 8.88 ± 4.00 (1–19) | 14.20 ± 7.46 (9–27) | NA | 0.005** |
| UPDRS II, mean ± SD (range) | 11.00 ± 5.42 (1–28) | 15.79 ± 5.79 (7–31) | 35.40 ± 6.47 (26–42) | NA | < 0.001*** |
| UPDRS III, mean ± SD (range) | 31.85 ± 14.05 (7–65) | 45.85 ± 12.67 (20–78) | 88.60 ± 23.30 (60–116) | NA | < 0.001*** |
| LEDDs (mg/d), mean ± SD (range) | 315.48 ± 268.20  (0–1250) | 480.61 ± 433.68  (0–2060) | 550.00 ± 274.43  (200–925) | NA | 0.067 |
| MMSE, mean ± SD (range) | 25.06 ± 4.41 (14–30) | 23.24 ± 7.00 (6–29) | 21.80 ± 5.85 (13–28) | NA | 0.257 |
| PRR14, ng/ml, median (QL–QU) | 36.52(21.55–64.16) | 37.41(29.69–68.12) | 40.98(21.49–67.99) | 30.26(21.22–43.45) | 0.835 |
| VCAM-1, pg/mL, median (Q_L_–Q_U_) | 1188000.00  (795599.27  –1443700.00) | 1045950.00  (764176.10  –1243925.00) | 1608500.00  (973051.77  –2322100.00) | 440188.30  (305452.80–  890782.00) | 0.036* |

The severity of the disease was assessed by modified H&Y stage when patients were “off state”. PD patients with H&Y stage 1.0–2.0 were recognized mild groups. H&Y stage 2.5–3.0 were divided into moderate groups, and H&Y stage 4.0–5.0 were advanced groups. Then we demonstrated that serum VCAM-1 levels were linked with the severity of the disease. Multi-group continuous variables in normal distribution and homogeneity of variance were compared by using the ANOVA, otherwise, the Kruskal–Wallis test were performed. ** P < 0.05; **P < 0.01; ***P < 0. 001.*

**Abbreviation:** PD, Parkinson's disease; HCs, healthy controls; AAO, age at onset; UPDRS, Unified Parkinson Disease Rating Scale; H&Y, Hoehn and Yahr scale; LEDDs, levodopa-equivalent daily doses; MMSE, Mini-Mental State Examination; PRR14, proline-rich protein 14; VCAM-1, vascular cell adhesion molecule-1; NA, not available; n, number.

**Supplementary Table 4.**  Clinical features according to H-Y stage in patients with PD

| Variable | **P value** | | | | | |
| --- | --- | --- | --- | --- | --- | --- |
|  | Mild vs  Moderate | Mild vs  Sever | Mild vs  Control | Moderate  vs Sever | Moderate vs Control | Sever vs  Control |
| VCAM-1 | 0.256 | 0.034* | NA | 0.011* | NA | NA |
| Duration of PD | 0.024 | 0.051 | NA | 0.788 | NA | NA |
| UPDRS I | 0.054 | 0.028* | NA | 0.441 | NA | NA |
| UPDRS II | < 0.001*** | < 0.001* | NA | 0.044* | NA | NA |
| UPDRS III | < 0.001*** | < 0.001*** | NA | <0.001*** | NA | NA |

LSD–t or Kruskal–Wallis test was used for pairwise comparisons after the multiple group comparison for mild, moderate, and the sever groups in PD patients. We found that mild PD group had less serum VCAM-1 levels than sever PD group. **P < 0.05; **P < 0.01; ***P < 0.001.*

**Abbreviation:** PD, Parkinson's disease; VCAM-1, vascular cell adhesion molecule-1; UPDRS, Unified Parkinson Disease Rating Scale; LSD test, post-hoc test (LSD) test; NA, not available; n, number.

**Supplementary Table 5.** Serum factors and clinical features according to AAO variables in patients with PD

| **Variable** | **AAO < 55 (n = 42)** | **AAO ≥ 55 (n = 58)** | **p** |
| --- | --- | --- | --- |
| Gender, n (female/male) | 13/26 | 21/37 | 0.771 |
| LEDDs (mg/d), mean ± SD (range) | 438.10 ±404.69(0–2060) | 340.86 ± 280.94 (0–950) | 0.299 |
| H&Y, mean ± SD (range) | 1.90 ± 0.66 (1–3) | 2.23 ± 0.95 (1–5) | 0.173 |
| MMSE, mean ± SD (range) | 26.07 ± 4.78 (8–30) | 23.02 ± 5.66 (6–30) | < 0.001*** |
| PRR14, ng/ml, median (QL–QU) | 34.81(20.10–63.74) | 38.62(23.73–64.58) | 0.519 |
| VCAM-1, pg/mL, median (QL–QU) | 1008733.00  (765843.39–1374650.00) | 1210100.00  (827983.01–1688550.00) | 0.104 |

AAO-related subgroup was divided according to the median of AAO. We found that VCAM-1 and PRR14 were irrelevant to AAO. Categorical variables were compared by using the Pearson Chi–square test. Continuous variables in normal distribution and homogeneity of variance were compared by using the two-sample t test, otherwise, the Mann–Whitney test were performed. ** P < 0.05; **P < 0.01; ***P < 0.001.*

**Abbreviation:** PD, Parkinson's disease; AAO, age at onset; H&Y, Hoehn and Yahr scale; LEDDs, levodopa-equivalent daily doses; MMSE, Mini-Mental State Examination; PRR14, proline-rich protein 14; VCAM-1, vascular cell adhesion molecule-1; n, number.

**Supplementary Table 6.** Serum factors and clinical features according to duration of PD variables in patients with PD

| **Variable** | **Duration of PD < 5 (n = 56)** | **Duration of PD ≥ 5 (n = 44)** | **p** |
| --- | --- | --- | --- |
| AAO, y, mean ± SD | 56.16 ± 8.15 | 52.98 ± 9.86 | 0.126 |
| LEDDs (mg/d), mean ± SD (range) | 280.71 ± 252.90 (0–1130) | 510.23 ± 392.61 (0–2060) | 0.001*** |
| H&Y, mean ± SD (range) | 1.78 ± 0.72 (1–4) | 2.50 ± 0.85(1–5) | < 0.001*** |
| MMSE, mean ± SD (range) | 24.25 ± 5.48 (6–30) | 24.36 ± 5.58 (7–29) | 0.769 |
| PRR14, ng/ml, median (QL–QU) | 34.08 (22.75–63.74) | 41.02 (22.63–68.93) | 0.259 |
| VCAM-1, pg/mL, median (QL–QU) | 1034300.00  (772946.10–1450600.00) | 1188000.00  (848532.90–1445600.00) | 0.525 |

Subgroup analyses uncovered that serum PRR14 and VCAM-1 levels were irrelevant to the duration of PD. Continuous variables in normal distribution and homogeneity of variance were compared by using the two-sample t test, otherwise, the Mann–Whitney test were performed. ** P < 0.05; **P < 0.01; ***P < 0.001.*

**Abbreviation:** PD, Parkinson's disease; AAO, age at onset; H&Y, Hoehn and Yahr scale; LEDDs, levodopa-equivalent daily doses; MMSE, Mini-Mental State Examination; PRR14, proline-rich protein 14; VCAM-1, vascular cell adhesion molecule-1; n, number.

**Supplementary Table 7.** Serum factors and clinical features according to clinical phenotypes in patients with PD

| **Variable** | **PD patients (n = 100)** | | |  |  |
| --- | --- | --- | --- | --- | --- |
|  | **TD (n = 40)** | **ID (n = 17)** | **PIGD (n = 43)** | **HCs**  **(n = 100)** | **p** |
| Age y, mean ± SD | 58.88 ± 7.81 | 59.16 ± 8.89 | 59.94 ± 11.88 | 57.44 ± 7.36 | 0.787 |
| AAO, y, mean ± SD | 54.70± 8.54 | 54.49 ± 8.72 | 55.59 ± 11.26 | NA | 0.747 |
| Duration of PD, y, mean ± SD (range) | 4.20 ± 3.14 (1–17) | 4.70 ± 3.30 (1–15) | 4.35 ± 3.04 (1–13) | NA | 0.759 |
| UPDRS I, mean ± SD (range) | 6.85 ± 4.34 (0–19) | 9.53 ± 6.40 (1–27) | 7.88 ± 6.34 (1–22) | NA | 0.158 |
| UPDRS II, mean ± SD (range) | 12.35 ± 5.56 (1–29) | 15.28 ± 9.66 (2–42) | 13.47 ± 6.51 (3–28) | NA | 0.719 |
| UPDRS III, mean ± SD (range) | 37.53 ± 14.77 (8–65) | 42.35 ± 23.15 (7–116) | 35.82 ± 16.97 (9–70) | NA | 0.649 |
| LEDDs (mg/d), mean ± SD (range) | 336.38 ± 384.34  (0–2060) | 424.77 ± 298.67  (0–1000) | 379.41 ± 333.02  (0–1130) | NA | 0.207 |
| H&Y, mean ± SD (range) | 1.90 ± 0.68 (1–3) | 2.31 ± 1.02 (1–5) | 2.00 ± 0.41 (1–3) | NA | 0.260 |
| MMSE, mean ± SD (range) | 24.00 ± 5.94 (6–30) | 24.19 ± 5.72 (7–29) | 25.29 ± 3.70 (15–29) | NA | 0.980 |
| PRR14, ng/ml, median (QL–QU) | 36.55(21.09–48.73) | 62.83(26.62–87.04) | 34.81(22.70–58.22) | 30.26(21.22–43.45) | 0.217 |
| VCAM-1, pg/mL, median (Q_L_–Q_U_) | 965640.60  (796792.36–  1296925.00) | 1312000.00  (985263.84–  1621450.00) | 1047900.00  (737847.16–  1454150.00) | 440188.30  (305452.80–  890782.00) | 0.110 |

The calculation of Movement Disorder Society-Unified Parkinson’s Disease Rating Scale (MDS-UPDRS) was used to define tremor dominant (TD), postural instability/gait difficulty (PIGD), and Indeterminate (ID) phenotypes of PD patients. Following empirical classification algorithms, the resultant ratio of the mean UPDRS tremor scores (8 items, 0–4 score for each item) to the mean UPDRS PIGD scores (5 items, 0–4 score for each item) were developed to define TD patients (ratio ≥ 1.15), PIGD patients (ratio ≤ 0.90), and indeterminate patients (ratios > 0.90 and < 1.15). Specially, if the numerator of the ratio was zero, and the denominator was positive, then these patients were defined as PIGD. Conversely, patients who had a positive numerator with a zero in the denominator were classified. And if both the numerator and denominator of the ratio were zero, the patient was pertinent to ID phenotype. But we found that discrete clinical phenotypes were irrelevant to PRR14 or VCAM-1. Multi-group continuous variables in normal distribution and homogeneity of variance were compared by using the ANOVA, otherwise, the Kruskal–Wallis test were performed. ** P < 0.05; **P < 0.01; ***P < 0.001.*

**Abbreviation:** PD, Parkinson's disease; HCs, healthy controls; PIGD, postural instability/gait difficulty; ID, indeterminate; TD, tremor dominant; AAO, age at onset; MDS-UPDRS, Movement Disorder Society-Unified Parkinson’s Disease Rating Scale; H&Y, Hoehn and Yahr scale; LEDDs, levodopa-equivalent daily doses; MMSE, Mini-Mental State Examination; PRR14, proline-rich protein 14; VCAM-1, vascular cell adhesion molecule-1; NA, not available; n, number.

# Supplementary Figures


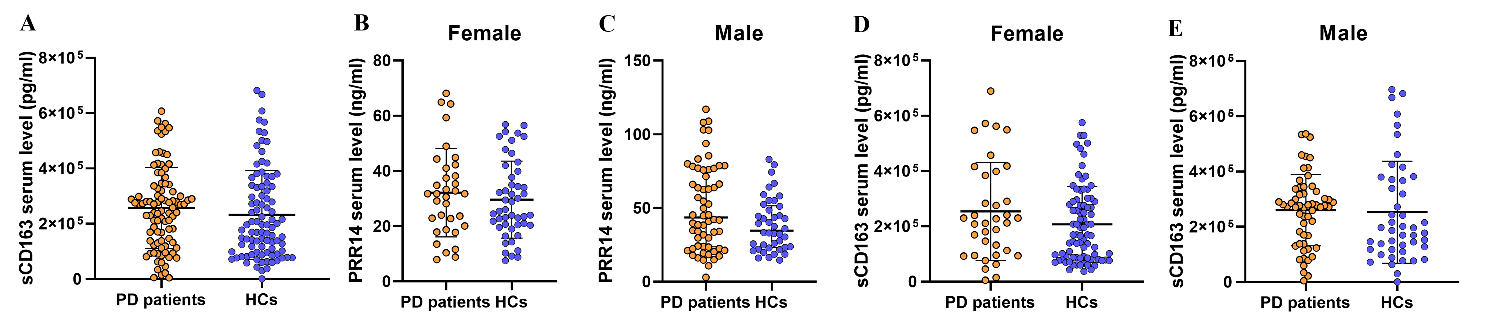


Fig. S1 For the alteration of PD patients and HCs groups, no significance was found in the serum sCD163 levels between two groups (A). For sex subgroup analysis, PRR14 and sCD163 serum levels were observed without the sex differences among the female (B and D) and male subgroups (C and E). Mann–Whitney U test.

**Abbreviation**: PD, Parkinson's disease; PRR14, proline-rich protein 14; sCD163, soluble CD163.
